# Participatory Methods to Engage Autistic People in the Design of Digital Technology: A Systematic Literature Review

**DOI:** 10.1007/s10803-023-06015-5

**Published:** 2023-05-30

**Authors:** Rachael Maun, Marc Fabri, Pip Trevorrow

**Affiliations:** 1 Leeds School of Arts, Leeds Beckett University, Leeds, LS1 3HE UK; 2 School of Built Environment, Engineering and Computing, Leeds Beckett University, Leeds, LS1 3HE UK

**Keywords:** Participation, Co-Design, User-centred Design, Technology, Engagement, Autistic Strengths

## Abstract

**Purpose:**

Many technology designers strive to involve end users in the design process, aiming to produce better outcomes. However, designers may struggle to engage autistic users effectively due to a lack of understanding of autistic characteristics and preferences. This systematic literature review aimed to identify how autistic adolescents and adults can best be engaged in effective participatory design activities.

**Methods:**

Seven databases were searched for articles reporting technology design involving autistic people, returning 276 results. Using the PRISMA approach, 258 were excluded due to not meeting the inclusion criteria. The remaining 18 articles were then quality assessed.

**Results:**

A thematic analysis revealed five core themes: (1) Engagement: the importance of investment in, and ownership of, the design process to create engagement, (2) Relationships: building relationships through collaboration and careful facilitation, (3) Skills development: the development of personal skills such as teamwork, design and self-advocacy in order to grow confidence, (4) Structure: providing context and structure to make the design experience feel safe and predictable, and (5) Support: the importance of support and consideration of individual needs as well as discouraging negative behaviours.

**Conclusion:**

Valuing participants as equal partners in design emerged as an, arguably, universal principle. The specific needs and preferences of autistic people must be understood and respected by designers. This goes beyond obvious accommodations such as providing a quiet and safe environment, and requires a deeper, more personal engagement with the individuals and their interests. We provide tangible recommendations for increasing engagement and aiding the design process.

## Introduction

Participatory design (PD) has been a focal point of many scholarly articles and attributed to the success of design firms such as IDEO and Continuum (Continuum, 2022; IDEO, 2022). Companies such as these specialise in the innovation of new products, services and experiences derived from user-centred design methods.

Participatory design, often used interchangeably with the terms co-design, co-creation and human-centred design aims to involve the user actively in the design process from conception of an idea through to prototype testing at the end of the design process (Simonsen & Robertson, 2012). There is no agreed definition of PD, yet there is some consensus on the core concepts that underpin it (Greenbaum & Loi, 2012; Luck, 2018): equalising power dynamics; using democratic practices; working with people in their environment; fostering mutual learning; using methods that allow people to design by doing and to express their needs, visions and ideas; and providing alternative visions about technology.

In this paper, we investigate how autistic people can best be engaged with the participatory design of digital technology that is meant to be beneficial to them. Through a systematic literature review, we explore the methods researchers employed when conducting participatory design, and how successful these were. The research was guided by two research questions;

- RQ1: What aspects of participatory design are the most engaging for autistic participants?
- RQ2: What affects the effectiveness of participatory design with autistic participants?

## Defining the Target Group

Autism is a developmental condition, characterised by difficulties with reciprocal social interactions, restricted or repetitive patterns of behaviour and routines, as well as atypical or excessive interests (WHO, 2020). These difficulties vary depending on the individual, and many autistic people are considered to have a ”spiky”, or uneven, set of abilities and capacities (Milton, 2012). To illustrate this, Milton posits that verbal autistic people are often incorrectly assumed to be capable in areas in which they struggle, whilst those with less verbal skills are often incorrectly assumed to be lacking in strengths or potential.

This may impact the involvement of autistic people in PD activities, as their capabilities may not be clear to designers or researchers. Likewise, limitations and support needs may be misunderstood or remain unnoticed. This could prevent autistic people from fully engaging in PD and clearly stating their needs, (e.g., a preference for structure and predictability) (Goris et al., 2020). Difficulties with Executive Functioning (EF), which relates to cognitive processes required to plan and perform complex tasks, abstract reasoning and the use of working memory (Dijkhuis, 2020) may further hinder active participation. Another relevant autism characteristic is weak central coherence (WCC) which relates to difficulties with generalising situations and drawing out meaning from detail (seeing the “bigger picture”), instead focusing extensively on small details (Happé, 2021). The often-speculative nature of PD methods, involving “blue sky” thinking and the consideration of several prototype options simultaneously, may also present challenges here. Therefore, established formats to conduct PD activities and the roles typically assumed during such activities need to be re-examined for this user group, as previously argued for by Satterfield & Fabri, (2019).

The use of PD with autistic people has grown over recent years, with autistic children between the ages of 8 and 12 being the most often researched group (Börjesson, 2015). This has generated a variety of new technologies, with outputs ranging from learning aids (Guldberg, 2017), social communication tools (Harrold, 2014; Abdullah & Brereton 2017), mental health aids (McGowan et al., 2017), to language tools (Plaisted Grant, 2019).

However, there is a need for a systematic literature review that considers PD with autistic adolescents (12+) and adults, a group that has been ignored thus far; existing reviews have focussed predominantly on developmentally diverse children in general (e.g. a review into participatory design with children with ADHD, autism, cerebral palsy and intellectual disabilities) (Börjesson, 2015). The decision to focus on this target group also fits within literature on cognitive development, that states that abstract and complex thinking does not occur until early adolescence, or from around the age of 12 onwards(Inhelder & Piaget, 1958; Goldman, 1965; Benton & Johnson, 2014) argue that abstract thinking is a core skill needed in the PD process, aiding idea generation. .

## Materials and Methods

### Search Strategy

In accordance with PRISMA guidelines (PRISMA, 2015), a systematic search of the literature on the use of participatory design with autistic people was conducted. Seven databases were searched (ACM, ERIC, MEDLINE, PsycINFO, Academic Search Complete and Scopus), using the following search syntax:

*(autism OR autistic OR ASC OR ASD) AND (“participatory design” OR “co-design” OR “co-creation” OR “design thinking” OR “human-centred design”)*

The reference lists of all eligible papers were also searched. Searches were limited to English language, peer-reviewed papers from 1st January 2004 to 20th October 2021. The review was restricted to this period as searches prior to 2004 yielded few to no results, this was also the case in the review by (Börjesson, 2015), into participatory design with developmentally diverse children.

**Fig. 1:**
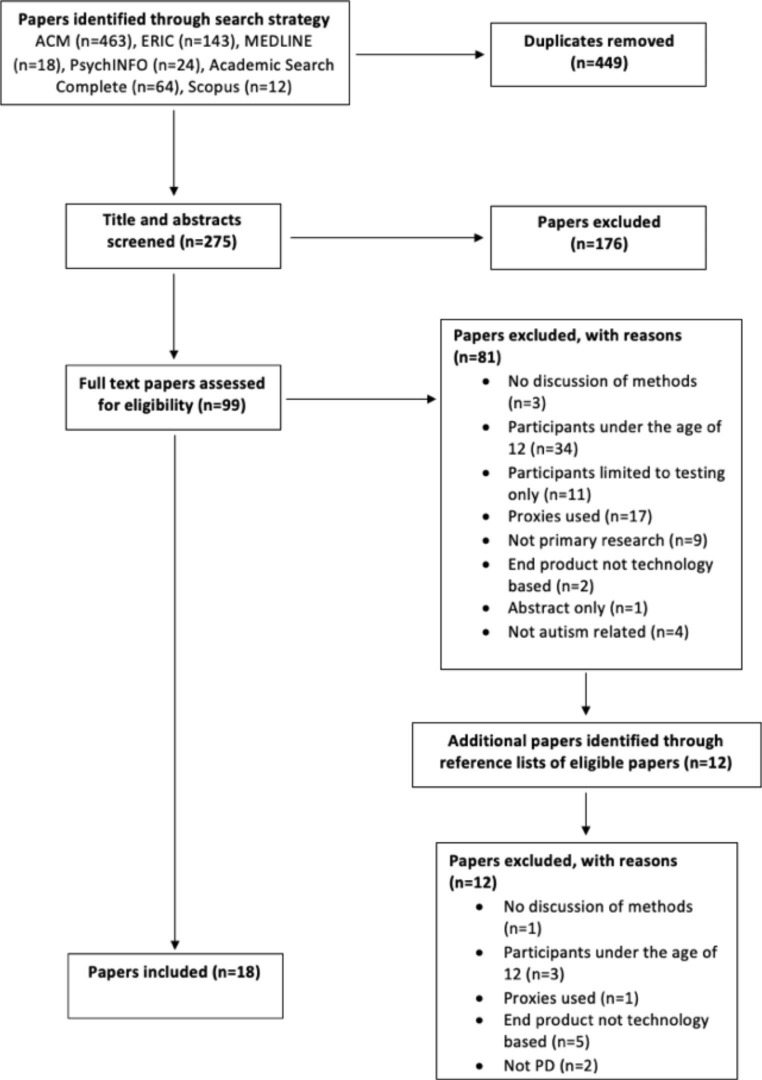
PRISMA diagram of the number of papers present at each stage of the systematic review process

Details on the number of papers present at each phase of the review process can be seen in the PRISMA Flow Diagram in Fig. 1. This diagram is a graphical representation of the number of papers identified in the systematic search and details the numbers of papers included at the title and abstract screening, full-text screening, and systematic review phases. It also details the numbers of duplicates removed and papers excluded and the reasons for exclusion of full-text papers.

### Selection Criteria

Papers were deemed eligible for inclusion if they examined the use of participatory design methods in technology design with people diagnosed or identifying as autistic, where the technologies are to be used by autistic people. For the purpose of this review, the following definitions will be used; participatory design is defined as the active involvement of autistic people in the conception or design of digital technology, where end users are not simply inspiring the designers or providing feedback, they are active contributors of design ideas and decision making. Participatory design methods are defined as the tools, strategies and activities that allow active involvement of the end users. Technology is defined as any piece of software, app, website, device, or service that is created or adopted for use by autistic people. Table 1 shows detailed information about inclusion and exclusion criteria.

**Table 1:** Inclusion and Exclusion Criteria

Inclusion Criteria	Exclusion Criteria
• Includes autistic participants • Makes use of participatory design/co-design etc. • Evaluation of discussion has been carried out on the methods employed • If not all participants are autistic, results are identifiable as relevant to autistic people	• Proxies are used • Autistic people’s involvement is limited to giving feedback on a prototype (or other methods which do not fully utilise participatory design) • Autistic people are not end users of the technology • End product is not a technology-based product • Participants under the age of 12

### Data Extraction and Synthesis

Screening of titles and abstracts of eligible papers was undertaken by three reviewers (< author 1>, <author 2 > and < author 3>). Full texts of papers remaining after the title and abstract screening were then read by the same reviewers and agreement was reached to exclude further papers that did not meet inclusion criteria. For all stages, any differences in opinion were resolved through consensus or discussion with all reviewers, based on the inclusion/exclusion criteria identified in Table 1. The reasons for exclusions at the full-text stage were recorded (see Fig. 1). The following was extracted from each paper: author, study design, sample size, methods used, and technology developed, as shown in Table 2.

**Table 2:** Overview of Included Studies

Author (Ref)	Study Design	Sample Size	PD Methods Used	Proxies used?	Technology Developed
Altizer Jr., (2018)	Qualitative	15–20	Presentations, discussions, surveys, interviews, prototype testing	Yes – parents (in final focus group)	Virtual reality system
Anthony, (2012)	Qualitative	12 (4 ASC)	Discussions, surveys, prototype testing	No	Application
Benton, (2012)	Mixed methods	6	Discussions, demonstrations, storyboards, surveys	Yes - Teachers	Game
Benton and Johnson, (2014)	Mixed methods	16 (6 ASC)	Discussions, demonstrations, developing prototypes, prototype testing, surveys	Yes – teachers	Game
Bossavit and Parsons (2016a)	Qualitative	20 (7 ASC)	Discussions, brainstorming, prototype testing	Yes – teachers and teaching assistants	Game
Bossavit and Parsons (2016b)	Qualitative	20 (7 ASC)	Demonstrations, discussions, prototype testing	Yes – teachers and teaching assistants	Game
Cascio, (2020)	Qualitative	7	Presentations, discussions	No	Bio-music
Cha, (2021)	Mixed Methods	8	Discussions, brainstorming, drawing, diaries, interviews, prototype testing	No	Voice-based conversational agent
Constantin, (2017)	Qualitative	15 (11 ASC)	Brainstorming, drawing	Yes – teaching assistant	Reward system
Fabri et al. (2016)	Qualitative	11	Discussions, demonstrations, brainstorming, surveys, prototype testing	Yes – one parent	Online toolkit
Grond, (2019)	Qualitative	7	Focus groups, discussions, demonstrations, surveys, prototype testing	No	Bio-music
Kim, B., (2020)	Mixed methods	229 surveys 10 interview (6 ASC) 14 PD (2 ASC)	Surveys, interviews, prototype testing	Yes – parents and therapists	Game
Magkafa et al. (2021)	Qualitative	13	Presentations, brainstorming, prototype development	Yes - teachers	Museum interface
Rapp, (2019)	Qualitative	5 PD 38 interviews 8 evaluations	Interviews	No	Interactive map
Simm, (2016)	Qualitative	13 (7 ASC)	Discussions, brainstorming, prototype development, prototype testing	Yes – support workers	Digital health technology
Sturm, (2019)	Qualitative	14 (10 ASC)	Brainstorming, prototype testing, interviews	No	Game
Zhu et al. (2019)	Qualitative	6	Discussions, storyboards, prototype development	Yes - parents	Social networking platform
Zhu et al. (2022)	Qualitative	6	Discussions, storyboards, prototype development, prototype testing	Yes - parents	Social networking platform

All data were analysed using the same synthesis method; thematic analysis as developed by (Braun & Clarke, 2012), as literature states that this type of synthesis is ideal for interpreting evidence on a particular topic or question and allows for the synthesis of different data types (quantitative and qualitative) and is traditionally used in systematic reviewing (Mays et al., 2005; Thomas & Harden, 2008).

Braun and Clarke’s (2012) approach to thematic analysis was undertaken by < author 1 > with an independent review of themes by < author 2 > and < author 3>, followed by a group discussion to clarify and refine interpretations. First, <author 1 > familiarised themselves with the data, before moving on to generating initial codes on paper. After generating initial codes, data was input into NVIVO (Lumivero, 2023), where themes were established and then reviewed by < author 2 > and < author 3>, then codes and themes were defined and named by all researchers collaboratively.

### Quality Appraisal

All 18 papers were quality appraised using the Mixed Methods Appraisal Tool (MMAT) (Hong, et al., 2018). The MMAT was specifically designed for use in systematic reviews in which a mixture of methods were used and allows for the appraisal of quantitative, qualitative and mixed methods papers using one tool. The MMAT tool has proven to be both reliable and efficient, and has been used widely in other systematic literature reviews (Pace, 2012; McNicholl et al., 2021).

## Results

### Study and Sample Characteristics

Eighteen papers describing fifteen studies (3 papers built upon previous research from the same study) were deemed eligible for inclusion by all three reviewers (< author 1>, <author 2>, <author 3>), as listed in Table 2. The studies included qualitative (n = 15) and mixed methods (n = 3) designs. Though all papers followed a participatory design approach, a series of different methods were utilised across the different research papers. The most often used methods were discussions, with 13 of the papers utilising this method and prototype testing, which was used in 12 of the papers. The least used methods were diaries, which were only used in one paper, drawing which was used in 2 papers and storyboarding which was used in 3 papers. Other methods used include, presentations (n = 3), surveys (n = 7), interviews (n = 5), demonstrations (n = 5) and prototype development (n = 5).

Sample sizes varied depending on whether pre-workshop surveys/questionnaires or interviews were conducted, though in terms of PD workshops, sample sizes ranged from 6 to 20 participants. Some papers focussed on the input of autistic people alone, where others included other diagnoses, and some included parents, carers, teachers, healthcare professionals etc.

### Quality Appraisal

All 18 papers were quality appraised using the Mixed Methods Appraisal Tool (MMAT) by all three reviewers, primarily < author 1 > and < author 2>, with < author 3 > reviewing the quality where disagreements occurred. The quality appraisal of included papers is outlined in Table 3.

**Table 3:** Quality Scores for Included Studies using Mixed Methods Appraisal Tool (MMAT)

Author (Ref)	Study Design	Criteria Met	Criteria Not Met/Cannot Tell	Overall MMAT Score (%)
Altizer Jr., (2018)	Qualitative	1.1	1.2, 1.3, 1.4	25%
Anthony, (2012)	Qualitative	1.1, 1.3, 1.4	1.2	75%
Benton, (2012)	Mixed methods	1.1, 1.2, 1.3, 1.4, 4.1, 4.2, 4.3,4.4, 5.1, 5.2	5.3	90%
Benton and Johnson, (2014)	Mixed methods	1.1, 1.2, 1.3, 1.4, 4.1, 4.2, 4.3, 4.4, 5.1, 5.2, 5.3		100%
Bossavit and Parsons (2016a)	Qualitative	1.1, 1.3 1.4	1.2	75%
Bossavit and Parsons (2016b)	Qualitative	1.1, 1.4	1.2, 1.3	50%
Cascio, (2020)	Qualitative	1.1, 1.3, 1.4	1.2	75%
Cha, (2021)	Mixed Methods	1.1, 1.2, 1.3, 1.4, 4.1, 4.2, 4.4, 5.1, 5.2, 5.3	4.3	90%
Constantin, (2017)	Qualitative	1.1, 1.2, 1.3	1.4	75%
Fabri et al. (2016)	Qualitative	1.1, 1.3	1.2, 1.4	50%
Grond, (2019)	Qualitative	1.1, 1.2, 1.3, 1.4		100%
Kim B., (2020)	Mixed methods	1.3, 4.1, 4.2, 4.3, 5.3	1.1, 1.2, 1.4, 4.4, 5.1, 5.2	45%
Magkafa et al. (2021)	Qualitative	1.1, 1.2, 1.3, 1.4		100%
Rapp, (2019)	Qualitative	1.1, 1.2, 1.3	1.4	75%
Simm, (2016)	Qualitative	1.1, 1.3	1.2, 1.4	50%
Sturm, (2019)	Qualitative	1.1, 1.2, 1.3	1.4	75%
Zhu et al. (2019)	Qualitative	1.1, 1.2, 1.3, 1.4		100%
Zhu et al. (2022)	Qualitative	1.1, 1.2, 1.3. 1,4		100%

In terms of criteria, only criteria 1.1–1.4 for qualitative papers, criteria 4.1–4.4 for quantitative papers and 5.1–5.3 for mixed methods papers were used. These criteria are outlined in Table 3.

### Synthesis of Results

Using the process of thematic analysis (Braun & Clarke, 2012), 18 initial codes were identified through inductive coding. Upon investigating the codes further, three were removed as there was not enough meaningful data to support the codes and other codes were merged to form stronger codes. From these codes, five analytical themes were identified in the data (see Table 4). Each of the themes is reported in detail below.

**Table 4:** Development of codes and themes

1. Initial codes	2. Removed codes	3. Final themes after merge
• Strength based view • Empowerment/confidence • Individual needs/personalisation • Positive reinforcement/reassurance • Learning • Engagement/enthusiasm • Interest/enjoyment • Project investment • Context • Ownership • Support • Collaboration/teamwork • Visual aids • Structure • Environment • Motivation • Challenge assumptions • Rapport/relationships	• Strength based view • Motivation • Challenge assumptions	• Engagement • Relationships • Skills development • Structure • Support

### Theme 1: Engagement

Across the papers reviewed, participant engagement was observed in several different ways, namely, engagement and enthusiasm, interest and enjoyment and project investment.

Engagement and enthusiasm presented itself through the PD process in different areas, with researchers reporting positive signs of engagement (e.g., laughing and jumping upon completing tasks), and negative signs, with some participants withdrawing from the design process completely ((Bossavit & Parsons, 2016a). Increased engagement was observed in different ways throughout the different research projects, though some notable areas were the use of hands on PD methods in which participants could explore the technologies (Anthony, 2012; Grond, 2019), giving participants an opportunity to share their experiences and ask questions (Fabri et al., 2016; Magkafa et al., 2021), clearly communicating expectations of the participants and their roles within the workshops (Bossavit & Parsons, 2016a, b) and spending increasing amounts of time engaging with the study (Zhu et al., 2019, 2022).

Interest and enjoyment were also presented similarly, with participants enjoying aspects such as being given the opportunity to express their views (Anthony, 2012; Magkafa et al., 2021) and being part of a team (Benton & Johnson, 2014; Zhu et al., 2022).

A sense of ownership and a feeling of project investment were also core to the engagement of participants in the PD process. Participants were able to gain a sense of ownership during the design process when they were able to see their ideas implemented into tangible designs (Benton, 2012; Benton & Johnson 2014; Simm, 2016; Sturm, 2019; Zhu et al., 2019, 2022). Project investment occurred when participants were listened to and able to contribute to the workshops (Benton, 2012; Fabri et al., 2016; Altizer Jr., 2018; Grond, 2019) and had an opportunity to ask questions (Magkafa et al., 2021).

### Theme 2: Relationships

Relationships emerged as an important aspect of the PD process, with collaboration and facilitator rapport being observed within the papers.

Collaboration presented itself as a way in which participants were made to feel included in the design process (Anthony, 2012), were able to build on each other’s ideas (Benton, 2012) and were able to interact with other group members, including helping members with tasks as and when required (Benton, 2012; Cascio, 2020). Collaboration occurred when participants were given an opportunity to contribute to the design process flexibly (Cascio, 2020), engage with other participants within the workshops (e.g., with small talk) (Zhu et al., 2019) and - in cases of working with adolescents - collaboration occurred when it was initially scaffolded by workshop facilitators or other adults, with it then beginning to occur naturally as the workshops and relationships progressed (Benton, 2012; Benton & Johnson 2014).

Facilitator rapport describes the relationships developed between the workshop facilitators and workshop participants. Facilitator rapport was only reported in research papers where the workshop facilitators immersed themselves into the environment prior to the commencement of the design process (Zhu et al., 2019, 2022; Magkafa et al., 2021). This community immersion helped create a group identity (Zhu et al., 2022), and reduce anxiety and increase self-confidence in participants (Magkafa et al., 2021) who became familiar with the community in which the research was being conducted (Zhu et al., 2019, 2022; Magkafa et al., 2021).

### Theme 3: Skills Development

The third theme that arose was skills development, with confidence and self-advocacy skills, design skills and teamwork skills all appearing within the papers.

Confidence presented itself through increased contributions to the design activities(Anthony, 2012) and independent idea generation (Benton, 2012; Benton & Johnson 2014; Magkafa et al., 2021). In some studies confidence improved across the design process, with participants gradually feeling more empowered to contribute as their skills grew (Anthony, 2012; Benton, 2012; Magkafa et al., 2021; Zhu et al., 2022). This increased confidence generally occurred in participants after involvement in one or more iterations of the design process (Benton, 2012; Zhu et al., 2022). Confidence also increased in younger participants when adult support was present (Benton, 2012; Benton & Johnson 2014).

Design skills were also developed in the PD process, with participants having opportunities to practice different aspects of design including; idea generation (Benton, 2012; Grond, 2019), sharing ideas ((Bossavit & Parsons, 2016a), and software design (Zhu et al., 2022). This development of design skills allowed for mutual learning (Grond, 2019; Zhu et al., 2019, 2022), where participants learnt from the researchers in terms of design skills and how to contribute to the design process, and researchers were able to learn from the participants by gaining an understanding into their technology preferences.

As mentioned previously, collaboration occurred during the PD process, with participants building on each other’s ideas and interacting with other group members (Anthony, 2012; Benton, 2012). This collaboration also led to the development of team work skills, especially where adolescents were involved in the design process, with teachers reporting an increase in teamworking, something which does not always come naturally to autistic individuals (Benton, 2012; Benton & Johnson 2014).

### Theme 4: Structure

The fourth theme that arose through the thematic analysis was structure, with context, environment and workshop activities all appearing as important aspects.

Giving participants context for the technologies being designed positively affected how the design process progressed. When participants were presented with a clear idea of what was being designed, engagement increased (Bossavit & Parsons, 2016a; Magkafa et al., 2021), and if the technology being designed appeared relevant or interesting to participants, this also had a positive impact (Anthony, 2012; Benton, 2012; Bossavit & Parsons 2016b).

The environment in which the workshops were conducted also affected the design process, with a familiar and safe environment being commonly attributed to the success of PD sessions (Benton, 2012; Bossavit & Parsons 2016a>; Simm, 2016; Constantin, 2017; Zhu et al., 2019, 2022; Cascio, 2020; Magkafa et al., 2021). Other considerations that had a positive effect on the design process included a quiet environment (Benton, 2012; Benton & Johnson 2014) and hosting the workshops at regular or predetermined intervals (Benton, 2012; Benton & Johnson 2014; Simm, 2016). On the other hand, facilitating the workshops in a room where distractions were possible (e.g., the opportunity to access computers, had a negative effect on the design process) (Bossavit & Parsons, 2016a, b), as did conducting the sessions in a room where sensory needs had not been considered (e.g., rooms with a strong smell), not enough room and bright lighting (Grond, 2019; Cascio, 2020).

The structure of workshop activities also had an impact on the design process, with well-structured activities benefitting younger participants (ages 12–13) (Benton, 2012; Benton & Johnson 2014), and freedom to explore benefitting older participants (UK college and university students, 16+) (Fabri et al., 2016). Having a consistent workshop structure was also found to be important (e.g., workshops taking place in the same room, at the same time each week) (Benton, 2012; Benton & Johnson 2014; Zhu et al., 2019, 2022; Magkafa et al., 2021), giving the participants structured roles within the activities (e.g., researcher, interviewer, tester etc.)(Benton & Johnson, 2014; Constantin, 2017) and introducing workshops, recapping previous sessions and offering a workshop plan (Benton, 2012; Fabri et al., 2016; Constantin, 2017; Zhu et al., 2019, 2022). A formal end to the PD process is also recommended, upon completion of all workshops (Grond, 2019).

### Theme 5: Support

The fifth theme from the data analysis was support, with adult support, flexibility and individual needs and visual and tangible aids emerging as codes.

The inclusion of adult support, when facilitating PD with adolescents, was a common occurrence, with the adults prompting participants, making suggestions, asking questions, re-engaging participants, discouraging negative behaviours, offering motivational support and explanations when needed, scribing for participants, facilitating interactions between participants and ensuring the wellbeing of all involved (Benton, 2012; Benton & Johnson 2014; Bossavit & Parsons, 2016a, b>; Constantin, 2017; Zhu et al., 2019, 2022; Magkafa et al., 2021). In some instances, this need for adult support reduced as younger participants increased in confidence, with adults being less involved as the workshops progressed (Benton, 2012; Benton & Johnson 2014; Bossavit & Parsons, 2016a).

The integration of flexibility and the consideration for individual needs also helped scaffold a supportive PD environment, with adult support (Benton & Johnson, 2014), adapted tools (e.g., braille) (Grond, 2019; Cascio, 2020), flexible approaches to contribution (Bossavit & Parsons, 2016a; Grond, 2019; Cascio, 2020; Magkafa et al., 2021) and opportunities for individual and small group workshops (Anthony, 2012; Cha, 2021) all increasing the contributions made within the workshops. It was also noted that individual needs should be considered in the design process, as not all users will use technologies in the same way and that this may affect the way participants contribute to the design process (Anthony, 2012; Altizer Jr., 2018; Cascio, 2020).

Visual and tangible aids also offered support in the design process, with demonstrations of existing technologies being useful in building context for the design process (Benton, 2012; Benton & Johnson 2014; Magkafa et al., 2021; Zhu et al., 2022), and demonstrations of developed prototypes being useful in eliciting feedback (Anthony, 2012; Benton, 2012; Bossavit & Parsons 2016a, b>; Simm, 2016). The integration of prototype testing for participants was also helpful with eliciting feedback with both working and paper prototypes being utilised in the various research studies. Other visual tools were also utilised in the studies, for example, visual schedules and screenshots/photographs which were useful in research projects with younger users (10–15) (Benton, 2012; Benton & Johnson 2014; Constantin, 2017; Magkafa et al., 2021), but were ignored in research with older participants (Bossavit & Parsons, 2016a, b).

## Discussion

This systematic review of 18 papers on participatory design with autistic adolescents and adults explored how this group can best be engaged in the design process. Five core themes emerged.

The first theme, *engagement*, relates directly to RQ1: What aspects of participatory design are the most engaging for autistic participants? Hands-on PD methods (Anthony, 2012; Grond, 2019), offering a space for sharing experiences (Fabri et al., 2016) and generating a sense of ownership and project investment (Altizer Jr., 2018; Sturm, 2019) were all found to increase the enjoyment and satisfaction of participants within the design process. were all found to increase the enjoyment and satisfaction of participants within the design process.

The second theme, *relationships, directly relates to* RQ2: What affects the effectiveness of participatory design with autistic participants?This theme showcased how developing strong group relationships can foster collaboration and in turn improve idea generation (Anthony, 2012; Benton, 2012). Difficulties with reciprocal social interactions (WHO, 2020) are counteracted by effective facilitator rapport and support. This can help develop a strong group identity (Magkafa et al., 2021; Zhu et al., 2022), which improves the effectiveness of the PD process and aids full participation. and helps full participation.

The third theme, *skills development*, focusses on the different skills that participants gained through the PD process, namely, increased confidence skills (Anthony, 2012), design skills (Benton, 2012; Grond, 2019) and teamwork skills (Benton, 2012; Benton & Johnson 2014). This increase in various skills was both empowering and satisfying for the participants involved. This addresses RQ1: What aspects of participatory design are the most engaging for autistic participants? In this context, it is essential that PD facilitators are aware of each participant’s strengths, interests and needs, or in other words the nature of their ”spiky” profile (Milton, 2012).

The fourth theme, *structure*, discussed how the structure of the workshops influenced the design process. Adolescents favoured structured tasks and activities (Benton, 2012; Benton & Johnson 2014), whilst adults enjoyed freedom to explore technologies on their own terms (Fabri et al., 2016). The context in which the research is situated in also needs to be explicit, as this gives participants a clear idea of their role in the design process, and in turn increases engagement (Bossavit & Parsons, 2016a; Magkafa et al., 2021). Consideration also needs to be given to the environment in which the sessions are being conducted, with familiar environments being best practice (Grond, 2019; Cascio, 2020), but when this is not possible, giving consideration to sensory disturbances (e.g., smell, lighting, space, airflow) (Benton, 2012; Benton & Johnson 2014). Similarly, workshops being scheduled for regular intervals is also beneficial to participants (Benton & Johnson, 2014; Magkafa et al., 2021; Zhu et al., 2022), appealing to a preference for routine and predictability (WHO, 2020). However, where this is not possible, organising workshops with advance notice is also positively received (Simm, 2016). This consideration for the structure of the PD both improves the effectiveness of the PD process and aids full participation, addressing both research questions. et al., 2016). This consideration for the structure of the PD both improves the effectiveness of the PD process and helps full participation, this addresses RQ1: What aspects of participatory design are the most engaging for autistic participants? and RQ2: What increases the effectiveness of participatory design with autistic participants?

The fifth theme, *support*, examined the different types of support offered in the PD workshops, namely; adult support (Benton & Johnson, 2014; (Bossavit & Parsons, 2016a), support for individual needs (Cascio, 2020) and support through visual and tangible aids (Anthony, 2012; Simm, 2016). PD activities requiring complex cognitive process require adequate scaffolding to account for difficulties with executive functioning (Dijkhuis, 2020). The same applies to activities requiring generalisations or the synthesising of information, to account for participants with weak central coherence (Happé, 2021). Examples of this support included, the integration and merging of multiple participant ideas being scaffolded by adults, the use of probing questions and prompts to promote new ideas, the use of adults to allow quieter members of the group to be heard, adults re-engaging adolescents in the design process when they have disengaged and offering words of affirmation and positive feedback when the adolescents’ were lacking confidence in their ideas (Benton, 2012; Benton & Johnson 2014; Bossavit & Parsons, 2016a, b>; Constantin, 2017; Sturm, 2019; Magkafa et al., 2021; Zhu et al., 2022). The various types of support utilised in the research papers increased the efficacy of the participatory design and helped with full participation in the workshops. This addresses RQ1: What aspects of participatory design are the most engaging for autistic participants? and RQ2: What affects the effectiveness of participatory design with autistic participants?, this addresses RQ1: What aspects of participatory design are the most engaging for autistic participants? and RQ2: What increases the effectiveness of participatory design with autistic participants?

When re-visiting the autism characteristics identified earlier, several recommendations can now be made, based on the themes identified and discussed above (see Table 5):

**Table 5:** Recommendations based on autism characteristics

Autism characteristic	Potential Impact	Recommendations
Capabilities misunderstood due to “spiky” profile	Designer may underestimate participants’ knowledge and their potential to engage or contribute. They may dismiss a participant’s unexpected contribution.	*1. Engagement*: Offer early hands-on PD activities with tangible outcomes to explore capabilities and increase investment. *2. Relationships*: Develop a strong relationship with the participant so that capabilities and interests are recognised and encouraged. *3. Skills development*: Pay attention to skills that may be developed during the PD process. These can empower the participant and increase engagement.
Limitations and support needs not clear or misunderstood, due to “spiky” profile	Participant is unable to contribute/engage effectively. Participant finds it difficult to express needs. Designer overestimates capability, creating uncomfortable interactions	*1. Engagement*: Run early test activities to gauge limitations and preferences. *2. Relationships*: Develop a strong relationship with the participant so that any support needs are understood early. *3. Skills development*: Adjust activities so that key skills can be developed during the PD process. *5. Support*: Respond to the support needs of each participant; adjust tasks, interactions and requests accordingly.
Difficulties with Executive Functioning	Participant has difficulty performing complex tasks, or with abstract reasoning.	*4. Structure*: Design activities to be clearly structured, with complex tasks broken down into sub-tasks. Make roles and expectation explicit. *5. Support*: Provide adequate scaffolding for complex tasks; offer positive affirmations to build confidence in participants’ abilities
Weak Central Coherence	Participant has difficulty with generalising from specific scenarios or tasks.	*3. Skills development*: Use scaffolding to develop generalisation skills gradually. *5. Support*: Use visual aids to illustrate/explain relationships and generalisations.
Abstract (“Blue Sky”) Thinking	Participant has difficulty with speculative thinking, or with considering several scenarios of use simultaneously.	*4. Structure*: Break down complex ideas into smaller parts, and deal with each one at a time. *5. Support*: Use prompts and probing questions to lead participants to new ideas.

## Conclusion

In conclusion, this systematic review highlights considerations for increasing the engagement of PD processes with autistic participants, as well as highlighting what helps full participation. Key findings include;

- The need for a safe space for participants to share ideas, which can be facilitated by giving participants an opportunity to express their views (Anthony, 2012; Magkafa et al., 2021).
- The need for a strong group rapport, which can be facilitated by interactions being initially scaffolded by adults, when working with adolescents, and allowing for engagement between participants when working with older participants (Benton & Johnson, 2014; Zhu et al., 2019).
- A need for context, so that participants are clear on their role in the design process (Magkafa et al., 2021).
- A consideration for structure, with younger participants thriving when structure is incorporated into the design process (Benton, 2012; Benton & Johnson 2014) and older participants preferring an opportunity to explore technologies and options freely (Fabri et al., 2016).
- The need for a quiet environment, with familiar environments being favoured where possible (Grond, 2019; Cascio, 2020).
- The benefits of adult support when working with adolescents particularly those aged 10–15 (Benton & Johnson, 2014; (Bossavit & Parsons, 2016b).
- A consideration for individual needs and preferences, especially in terms of alternative forms of contribution within the workshops (Grond, 2019; Cascio, 2020).
- The integration of tangible aids (e.g., working prototypes) (Anthony, 2012; Simm, 2016) and visual aids, when working with adolescents (Benton & Johnson, 2014; Magkafa et al., 2021).
- The consideration of special interests that some of the participants have, and incorporation of these into the PD activities, in order to increase engagement (Zhu et al., 2019, 2022).

### Limitations

This systematic literature review has several potential limitations that need to be mentioned. Firstly, there is a potential for studies with low quality to contribute little to the data synthesis, with better quality studies dominating contributions (Thomas & Harden, 2008). Secondly, thematic analysis often focuses on the similarities between papers, at the expense of acknowledging diverse data (Lucas, 2007). This was mitigated by allowing looking for nuances within each developed theme. Finally, there is the potential for bias despite all 3 authors having been involved in selecting relevant papers from search results, and then screening these papers for quality. We believe any such bias was minimised through the independent selection and coding process, and due to the differences in research backgrounds between coders.
